# Effects of nitrogen fertilization combined with subsurface irrigation on alfalfa yield, water and nitrogen use efficiency, quality, and economic benefits

**DOI:** 10.3389/fpls.2024.1339417

**Published:** 2024-01-29

**Authors:** Hongxiu Ma, Peng Jiang, Xiaojuan Zhang, Wenli Ma, Zhanhong Cai, Quan Sun

**Affiliations:** ^1^ College of Forestry and Prataculture, Ningxia University, Yinchuan, Ningxia, China; ^2^ Ningxia Reclamation, Agricultural, Forestry, and Animal Husbandry Technology Promotion and Service Center, Yinchuan, Ningxia, China

**Keywords:** multiple regression analysis, Medicago sativa L., fertilizer management, water productivity, crude protein content

## Abstract

Proper water and fertilizer management strategies are essential for alfalfa cultivation in arid areas. However, at present, the optimal amounts of subsurface irrigation and nitrogen (N) supply for alfalfa (*Medicago sativa* L.) cultivation are still unclear. Therefore, a field experiment was conducted in 2022 in Yinchuan, Ningxia, China, to explore the effects of different subsurface irrigation levels (W_1_, 50% of ET_C_ (crop evapotranspiration); W_2_, 75% of ET_C_; W_3_, 100% of ET_C_) and N application rates (N_0_, 0 kg/ha; N_1_, 75 kg/ha; N_2_, 150 kg/ha; N_3_, 225 kg/ha; N_4_, 300 kg/ha) on alfalfa yield, crop water productivity (CWP), N use efficiency (NUE), quality, and economic benefits. Besides, the least squares method and multiple regression analysis were used to explore the optimal water and N combination for alfalfa cultivation under subsurface irrigation. The results showed that the alfalfa yield, crude ash content, and partial factor productivity from applied N (PFPN) were the highest under W_2_ level, but there was no difference in PFPN compared with that under W_3_ level. The branch number (BN), leaf area index (LAI), yield, CWP, irrigation water productivity (IWP), crude protein content (CPC), and economic benefits increased and then decreased with the increase of N application rate, reaching a maximum at the N_2_ or N_3_ level, while the NUE and PFPN decreased with the increase of N application rate. Considering the yield, CWP, NUE, quality, and economic benefits, W_2_N_2_ treatment was the optimal for alfalfa cultivation under subsurface irrigation. Besides, when the irrigation volume and N application rate were 69.8 ~ 88.7% of ET_C_ and 145 ~ 190 kg/ha, respectively (confidence interval: 85%), the yield, CPC, and economic benefits reached more than 85% of the maximum. This study will provide technique reference for the water and N management in alfalfa cultivation in Northwest China.

## 1 Introduction

Currently, the demands for foods of animal origin rich in protein and nutrition (FAO, 2017) has increased like never before (National Research Council, 2015). Therefore, the cultivation of forage grasses is particularly important. Alfalfa, a homotetraploid perennial forage grass, is widely cultivated in the world due to its high yield, high protein content, and good palatability (Li and Brummer, 2012; Wagle et al., 2019). However, alfalfa growth requires large amounts of water, and frequent drought often leads to yield losses of alfalfa in arid areas (Lamm et al., 2012). Therefore, developing water-saving irrigation measures to improve alfalfa CWP and yield is very urgent (Oweis et al., 2004).

Subsurface drip irrigation provides water and nutrients directly to crop roots (Dukes and Schol berg, 2005). This technology saves water (about 20% ~ 30%) and fertilizer by reducing the loss of water and fertilizer, which is conducive to increasing crop yield and reducing cost in arid areas (Du et al., 2017). Previous studies have reported that subsurface drip irrigation can significantly enhance water production efficiency and alfalfa yield (Angold et al., 2015; Han et al., 2019). Besides, deep buried pipes does not affect mechanical harvesting operations, and there is no need to stop water and fertilizer supply one week before and after each cutting to suppress the mildew of alfalfa grass. This could prolong the growing period and increase yield.

Nitrogen fertilization is necessary for crop cultivation. Although alfalfa is a leguminous plant, the N fixed by rhizobia accounts for only 50% ~ 60% of alfalfa N requirement (De Oliveira et al., 2004). Therefore, it needs exogenous N supply. However, the optimal N application rate for alfalfa under subsurface irrigation remains unclear. Insufficient N supply cannot maintain the normal growth of alfalfa and affects CWP, yield formation, and quality, while excessive N supply may limit crop growth, reduce N utilization, and even cause environmental pollution (Liu et al., 2019). Therefore, the determination of optimal N application rate is very necessary (Delevatti et al., 2019; Sun et al., 2022). It should be noted that the N uptake in crops is also affected by soil moisture. Yin et al. (2012) reported that appropriate N application could improve alfalfa CWP and increase yield under deficit irrigation (80%). Xue et al. (2017) and Bu et al. (2014) pointed out that according to crop nutrient demand, the combination of irrigation and fertilization under subsurface irrigation can effectively inhibit vegetative growth. Therefore, it is necessary to consider the N application rate and the irrigation rate in an integrated manner.

At present, high irrigation and fertilization rates are widely used in local alfalfa cultivation, which increases production costs and groundwater contamination risk (Sha et al., 2021). Therefore, water and fertilizer reductions are very necessary. However, it is still unclear how to reduce subsurface drip irrigation rate and fertilization rate in alfalfa cultivation while maintaining high yield and economic benefits and what are the optimal ranges of irrigation and fertilization rates. This study hypothesized that moderately reducing irrigation and fertilization rates might yielded the optimal outcomes. To valid this hypothesis, in this study, in the arid region of Northwest China, the effects of different irrigation rates and N application rates on alfalfa growth, resource use efficiency, yield, quality, and economic benefits were explored. The objectives of this study were: (1) to compare the effects of different irrigation rates and N application rates on alfalfa growth, resource use efficiency, yield, quality, and economic benefits under subsurface drip irrigation; and (2) to determine the optimal irrigation and N application ranges that simultaneously maximize alfalfa growth parameters, resource use efficiency, yield, quality, and economic benefits under the premise of water and N reduction. This study will provide a technical reference for the water and fertilizer management for alfalfa cultivation in arid and semi-arid areas.

## 2 Materials and methods

### 2.1 Experimental site

The experiment was conducted in the Botanical Garden in Liangtian Town, Yinchuan, Ningxia, China (106°18′E, 38°40′N, a.s.l. 1100 m). The region has a temperate continental climate. The average annual temperature was 8.7°C, the average annual sunshine hour was 3032 hours, the frost-free period lasted 185 days, the average annual precipitation was 200 mm, and the average annual evaporation was 1694 mm (**Figure S1**). The soil type was sandy soil, with sand, silt, and clay accounting for 91.76%, 7.04%, and 1.20%, respectively. The soil bulk density was 1.47 g/cm^3^, the field capacity was 18.8% (g/g), and the wilting point was 8.9% (g/g). The soil organic matter content was 4.67 g/kg, the total N content was 0.31 g/kg, the available potassium content was 81.42 mg/kg, the available phosphorus content was 2.44 mg/kg, and the pH was 8.62.

### 2.2 Experimental design

Subsurface irrigation system was used in this study. The inner diameter of the pipes was 13 mm, and the wall thickness was 1.5 mm. Under the pressure of 0.06 MPa, the flow rate was 60-100 mL/(m·min). The pipe spacing was 80 cm, and the buried depth was 20 cm (Latorre et al., 2018).

The experiment employed a split-plot design, with irrigation volume (W_1_ (50% of ET_C_ (crop evapotranspiration)), W_2_ (75% of ET_C_), and W_3_ (100% of ET_C_)) as the main plot, and N application rate (N_0_ (0 kg/ha), N_1_ (75 kg/ha), N_2_ (150 kg/ha), N_3_ (225 kg/ha), and N_4_ (300 kg/ha)) as the subplot. Each group had three replicates/plots. The area of each plot was 12.5 m^2^ (2.5 m × 5 m). To prevent interference between treatments, a plastic film was vertically buried between plots (60 cm in depth).

**Figure 1:**
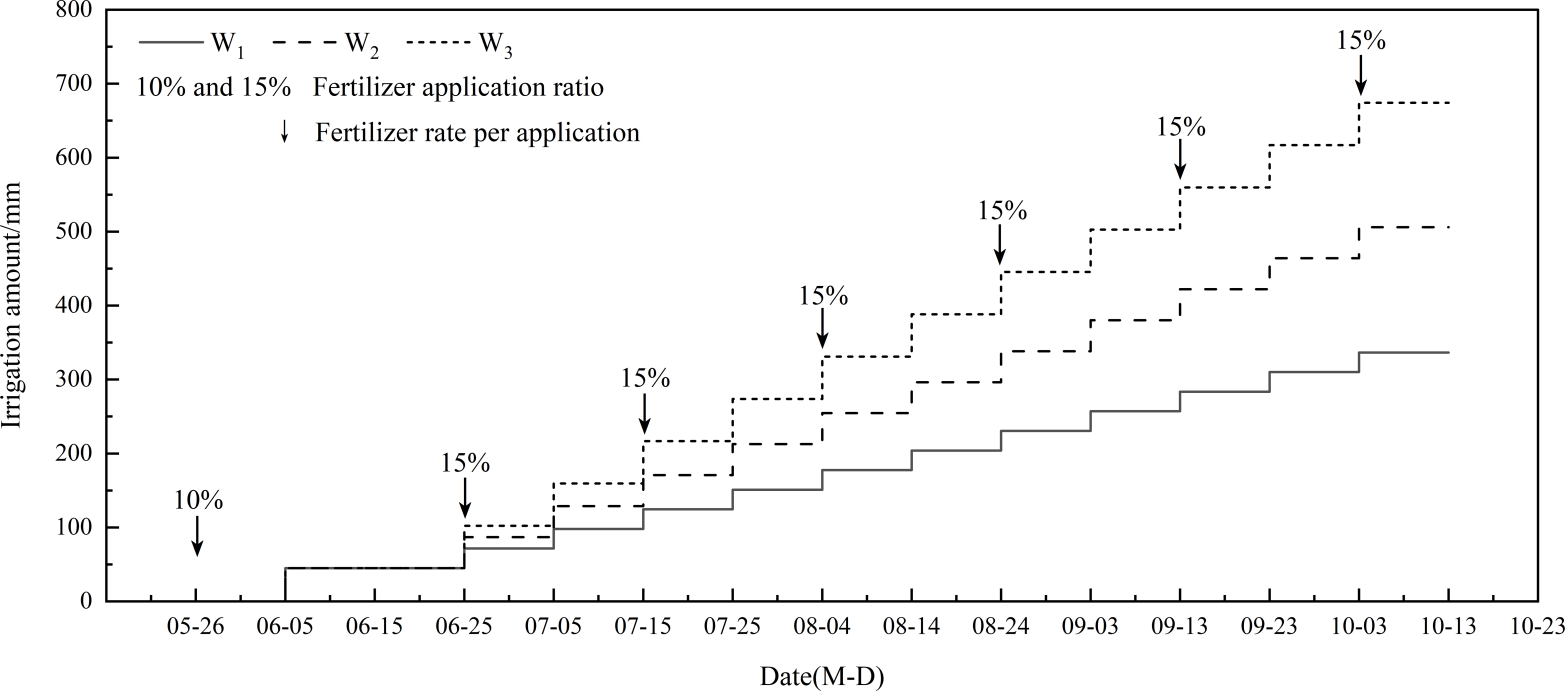
Details of irrigation and fertilization.

Before sowing, 10% of urea (N, 46%), 150 kg/ha of monoammonium phosphate (P_2_O_5_, 61%), and 120 kg/ha of potassium sulfate (K_2_O, 52%) were applied to the soil. After that (June 5, 2022), alfalfa seeds (variety “Magna Graze 401” (Canada)) were sown (15 kg/ha), with a sowing depth of 1 ~ 2 cm and a row spacing of 20 cm. Irrigation began on the day of alfalfa planting (June 5), with a volume of 45 mm. Then, irrigation was conducted every 10 days (12 times in total). The remaining urea was divided into six parts, and applied to the field through the irrigation system on June 25, July 15, August 4, August 24, September 13, and October 3 after completely dissolving in water (**Figure 1**). Other managements were the same as local practice. Alfalfa was cut two times in 2022 (August 10 and October 5).

### 2.3 Measurement methods

#### 2.3.1 Measurement of plant height, branch number, and leaf area index

At the initial flowering stage, 20 plants were randomly selected in each plot, and the vertical height after straightening was measured. Finally, the average value for each plot was calculated. Besides, the number of primary branches and leaves was counted (Wu et al., 2011). The length and width of the fully expanded top third leaf were measured with a vernier caliper, and the leaf area and LAI were calculated according to the methods of Hu et al. (2018).

#### 2.3.2 Measurement of CWP and IWP

The crop water productivity (CWP) and irrigation water productivity (IWP) was calculated using Equation (1) and Equation (2):


(1) CWP(kg/ha/mm)=Y/ET

where Y is the annual hay yield of alfalfa (kg/ha), and ET is the water consumption of alfalfa during the growing season (mm). The ET was calculated using the method of Allen et al. (1998). Due to the flat terrain and deep groundwater level in this area, groundwater recharge, surface runoff, and water infiltration are ignored.


(2) $$\text{IWP}\left(\text{kg}/{\text{m}}^{3}\right)=\text{Y}/\text{I}$$

where I is the total irrigation volume (mm).

Crop evapotranspiration (ET_C_) was calculated using Equation (3):


(3) $${\text{ET}}_{\text{C}}={\text{KcET}}_{0}$$

Where Kc is the crop coefficient based on FAO-56. The average crop coefficient of alfalfa was 0.87 in the first cut and 0.85 in the second cut (Zhang et al., 2016). ET_0_ is the evapotranspiration of reference crop (mm/d), calculated using Equation (4):


(4) $${\text{ET}}_{0}=\frac{0.408\Delta \left(R_{n}-G\right)+\gamma \frac{900}{T+273}U_{2}\left(e_{s}-e_{a}\right)}{\Delta +\gamma \left(1+0.34U_{2}\right)}$$

*Δ* is the actual vapor pressure (kPa), *Rn* is the net surface radiation (MJ/m^2^ d), *G* is the soil heat flux (MJ/m^2^ d), *γ* is the hygrometer constant (0.067kPa/°C), *T* is the average temperature (°C) at an altitude of 2 m, *U_2_* is the daily average wind speed at an altitude of 2 m, *e_s_* is the saturated vapor pressure (kPa), and *e_a_* is the actual vapor pressure (kPa). The meteorological data was obtained from the National Meteorological Science Data Center (http://data.cma.cn/).

#### 2.3.3 Measurement of NUE and PFPN

The nitrogen content in plants was determined by Kjeldahl method (Ferreira et al., 2015).

Plant nitrogen uptake (NU, kg/ha) was calculated using Equation (5):


(5) NU=TN×Y

where TN is the total nitrogen content of each organ of the plant.

The NUE (kg/kg) and PFPN (kg/kg) were calculated using Equation (6) and Equation (7):


(6) NUE=Y/NU

(7) PFPN=Y/FN

Where FN is the nitrogen application rate (kg/ha) during alfalfa growth period.

#### 2.3.4 Measurement of alfalfa yield and quality

At the initial flowering stage, 1 m^2^ (1 m×1 m) subplot was selected from each plot for cutting and weighed to obtain the fresh weight. Then, three fresh samples (about 300 g per sample) were collected, air-dried to the constant weight, and weighed. The fresh weight/dry weight ratio was calculated, to obtain the hay yield (Liu et al., 2015). After that, the alfalfa grass samples were crushed with a pulverizer (JFSO-480, Zhejiang Topunnong Technology Co) and passed through a 0.42 mm sieve. The crude ash content (ASH) was determined by high-temperature ignition, and the crude protein content (CPC) was determined by Kjeldahl method (Wen et al., 2018).

#### 2.3.5 Measurement of economic benefit (Eb)

The Eb (USD/ha) was calculated using Equation (8) (Zou et al., 2020):


(8) Eb=Gp−Wc−Fc−C

Where Eb is the economic benefit, Gp is the gross profit (USD/ha), Wc is the irrigation cost (USD/ha), Fc is the fertilizer cost (USD/ha), and C is the other costs (USD/ha).

#### 2.3.6 Comprehensive evaluation

To achieve the goals of high yield and high quality, the coupling effect of water and N on alfalfa was comprehensively evaluated based on yield, CWP, PFPN, CPC, and economic benefits. Based on the principle of least squares, the binary quadratic regression equation was established, with irrigation volume and N application rate as the independent variables, and yield, CWP, PFPN, CPC, and economic benefits as the dependent variables. The test data was analyzed using Mathematica 9.0 software to calculate the optimal irrigation and fertilization ranges when the indicators reached the maximum value.

### 2.4 Data analysis

Each measurement was repeated 3 times, and the average value was used for analysis. Analysis of variance was conducted using Excel 2007and SPSS 18.0. The ANOVA analysis was performed with the irrigation amount and fertilizer application rate as the main effects, and the interaction was also considered. Duncan’s new multiple range test method was used for multiple comparisons. Figures were drawn with Mathematica 9.0 and Origin 8.0.

## 3 Results

### 3.1 Plant height, branch number, leaf area index, and their relationships with yield

At the W_1_ and W_3_ levels, the plant height first increased and then decreased with the increase of N application rate. There was no difference in plant height between N_1_, N_2_, and N_3_ treatments, but the plant height in the N_1_, N_2_, and N_3_ treatments were significantly higher than that in the N_0_ and N_4_ treatments. At the W_2_ level, there was no difference in plant height between the five N treatments. Under the same N application rate, the plant height at the W_2_ level was 11.96 ~ 13.31% (first cut) and 5.60 ~ 5.87% (second cut) higher than that at the W_1_ and W_3_ levels, respectively. The W_2_N_3_ treatment had the maximum plant height for both cuts (**Table 1**).

**Table 1:** Plant height, branch number, leaf area index, and yield of alfalfa at different water and nitrogen supply conditions.

Treatment		Plant height (H cm)		Number of branches per plant		Leaf area index		Yield(t/ha)	
Irrigation	Fertilization	1st cut	2nd cut	1st cut	2nd cut	1st cut	2nd cut	1st cut	2nd cut
W_1_	N_0_	41.60d	40.67e	10.50c	10.66b	3.98g	4.35e	1.85i	2.31g
	N_1_	48.12ab	47.17abcd	10.42c	10.50b	4.63f	5.56d	2.46fg	2.97f
	N_2_	48.5ab	48.13abc	11.75ab	11.35ab	5.38e	6.31bc	2.69f	3.53e
	N_3_	49.2ab	48.33abc	11.83ab	11.17b	5.76de	6.76b	2.81f	3.85cde
	N_4_	41.10bc	41.47e	10.32c	10.67b	4.75f	5.38d	2.20h	3.03f
W_2_	N_0_	48.25ab	45.17cd	11.52abc	10.67b	4.96f	5.87cd	2.14h	3.55e
	N_1_	51.36ab	48.50abc	11.50abc	11.05b	6.19c	7.75a	3.60cd	4.10cd
	N_2_	53.33ab	49.03ab	12.17a	12.50a	7.57a	8.16a	4.29a	4.50ab
	N_3_	54.50a	50.44a	11.97ab	11.83ab	6.64b	7.76a	3.41de	4.21bc
	N_4_	51.70ab	46.69bcd	11.35abc	11.17b	5.96cd	6.75b	2.82f	4.13cd
W_3_	N_0_	45.33cd	44.70d	10.83bc	10.82b	5.70de	6.49b	3.18e	3.83de
	N_1_	47.50ab	46.25bcd	11.36abc	10.89b	6.06cd	7.86a	3.75bc	4.02cd
	N_2_	45.00ab	47.00bcd	11.83ab	11.62ab	6.67b	7.89a	3.92b	4.16cd
	N_3_	45.67ab	48.46abc	12.00ab	11.17b	6.28bc	7.91a	3.50cd	4.68a
	N_4_	40.63d	40.00e	11.33abc	10.65b	4.94f	5.85cd	1.97hi	3.67e
Significant level									
Irrigation		**	**	*	*	**	**	**	**
Nitrogen		**	**	**	**	**	**	**	**
Irrigation × Nitrogen		*	*	ns	ns	**	**	**	**

W_1_, 50% of ETC (crop evapotranspiration)); W_2_, 75% of ETC; W_3_, 100% of ETC; N_0_, 0 kg N ha; N_1_, 75 kg N ha; N_2_, 150 kg N ha; N_3_, 225 kg N ha; N_4_, 300 kg N ha. The same below. Different lowercase letters in the same column indicate significance at p< 0.05. *, p< 0.05; **, p< 0.01; ns, p > 0.05.

At the same irrigation level, the branch number first increased and then decreased with the increase of N application rate, and reached a maximum in the N_2_ or N_3_ treatments. The branch number in the N_2_ and N_3_ treatments were 6.91 ~ 8.23% (first cut) and 4.93% ~ 9.37% (second cut) higher than that in the N_0_, N_1_, and N_4_ treatments. At the same N application rate, there was no difference in the branch number in the two cuts between different irrigation levels. The W_2_N_2_ treatment had the highest number of branches (12.17 in the first cut and 12.50 in the second cut), and there was no significant difference between W_2_N_2_ and W_2_N_3_ treatments.

At the same irrigation level, the LAI first increased and then decreased with the increase of N application rate. The mean LAI in the N_1_, N_2_, and N_3_ treatments were 7.23 ~ 23.36% (first cut) and 15.11% ~ 25.50% (second cut) higher than that in the N_0_ and N_4_ treatments. At the same N application rate, the LAI at the W_2_ and W_3_ levels were significantly higher than that at the W_1_ level in the two cuts. The LAI in the W_2_N_2_ treatment reached a maximum of 7.57 and 8.16 in the first and second cuts, respectively (**Table 1**).

Under high irrigation volume and high N application rate conditions, alfalfa yield did not reach the maximum. However, the maximum occurred in the W_2_N_2_ (first cut) and W_3_N_3_ (second cut) treatment. At the same irrigation level, the yield first increased, reached a maximum in the N_2_ or N_3_ treatment, and then decreased, with the increase of N application rate. The maximal yield was 9.87-28.13% (first cut) and 9.06%-23.42% (second cut) higher than that in the N0, N1, and N4 treatments. At the same N application rate, the yield at the W_1_ level was lower than that at the W_2_ and W_3_ levels (**Table 1**).

**Figure 2:**
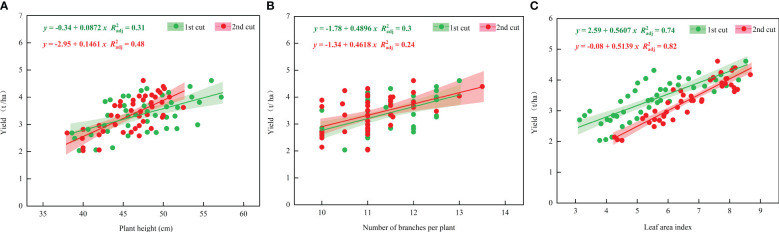
Relationship between alfalfa yield (Y) and growth indices (**A**, plant height; **B**, branch number; **C**, leaf area index).

The yield was positively correlated with plant height, branch number, and LAI. Especially, the correlation with LAI was the highest, with the R^2^_adj_ (adjusted coefficient of determination) of the first and second cut being 0.74 and 0.82, respectively (**Figure 2**).

### 3.2 The CWP and IWP of alfalfa under different water and nitrogen supply conditions

**Figure 3:**
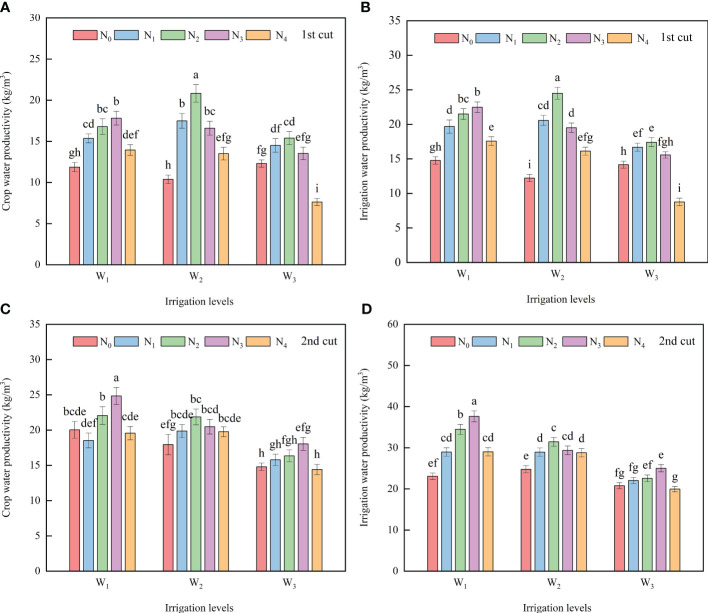
Effects of different irrigation volumes and nitrogen application rates on crop water productivity (CWP) **(A, C)** and irrigation water productivity (IWP) **(B, D)** of alfalfa. Different letters on top of the bar denote significant differences at *p* < 0.05 according to the LSD test. W1, 50% of ETC (crop evapotranspiration)); W2, 75% of ETC; W3, 100% of ETC; N0, 0 kg N ha; N1, 75 kg N ha; N2, 150 kg N ha; N3, 225 kg N ha; N4, 300 kg N ha.

The experimental results of the two cuts showed that the CWP increased first and then decreased with the increase of N application rate at the same irrigation level. At the W_1_ level, the CWP in the N_3_ treatment was 5.77 ~ 33.39% (first cut) and 11.17% ~ 25.42% (second cut) higher than that in the other N treatments. At the W_2_ level, the CWP in the N_1_, N_2_, N_3_, and N_4_ treatment was 23.12 ~ 50.17% (first cut) and 9.17% ~ 17.86% (second cut) higher than that in the N_0_ treatment. At the W_3_ level, there was no difference between N treatments, except for a significant decrease in CWP in the N_4_ treatment in the first cut compared with other N treatments. Under the same N application rate, except for the W_3_N_0_ treatment in the first cut, the yield at the W_3_ level was lower than that at the W_1_ and W_2_ level, and the CWP at the W_2_ level was 19.62% (first cut) and 20.55% (second cut) higher than that at the W_3_ level. The W_2_N_2_ treatment had the highest average CWP of the two cuts (21.35 kg/m^3^), and the W_3_N_4_ treatment had the lowest (11.02 kg/m^3^) (**Figures 3A, C**).

At the same irrigation level, the IWP first increased and then decreased with the increase of N application rate. At the W_1_ level, the IWP in the N_3_ treatment was 4.66 ~ 34.50% (first cut) and 5.95% ~ 29.59% (second cut) higher than that in the other N treatments. At the W_2_ level, the IWP in the N_1_, N_2_, N_3_, and N_4_ treatment were 23.82 ~ 49.88% (first cut) and 15.71% ~ 29.87% (second cut) higher than that in the N_0_ treatment. At the W_3_ level, there were no differences between N treatments, except for a decrease in IWP in the N_4_ treatment in the first cut compared with other N treatments. Under the same nitrogen application rate, except for N_0_, the IWP at the W_3_ level was lower than that at the W_1_ and W_2_ levels, and the IWP at the W_2_ level was 21.60% (first cut) and 20.66% (second cut) higher than that at the W_3_ level. The W_1_N_3_ treatment had the highest average IWP of the two cuts (26.69 kg/m^3^), followed by the W_2_N_2_ treatment (26.04 kg/m^3^), and the W_3_N_4_ treatment had the lowest (13.25 kg/m^3^) (**Figures 3B, D**).

### 3.3 The NUE and PFPN of alfalfa under different water and nitrogen supply conditions

**Figure 4:**
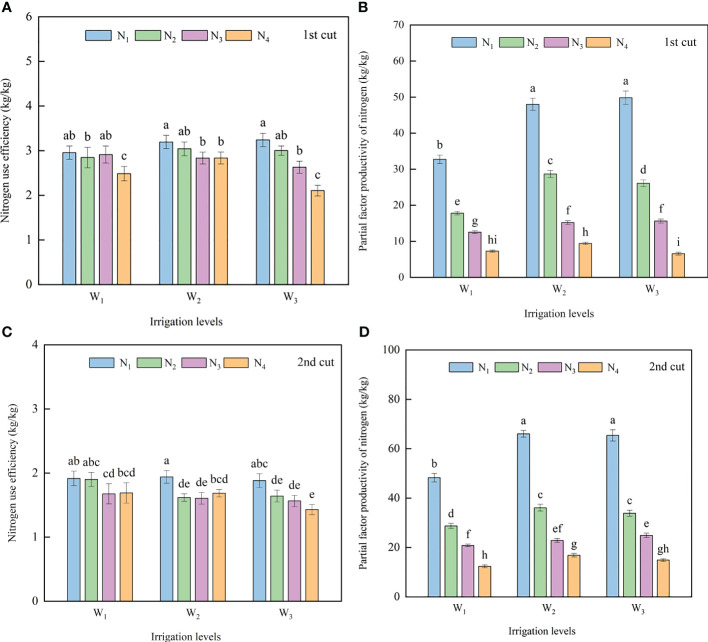
Effects of different irrigation volumes and nitrogen application rates on nitrogen use efficiency (NUE) **(A, C)** and partial factor productivity from applied N (PFPN) **(B, D)** of alfalfa. Different letters on top of the bar denote significant difference at *p* < 0.05 (LSD test). Different lowercase letters indicate significant differences between treatments at *p* < 0.05 (Tukey test). The processing abbreviations are the same as those described in **Figure 3**.

At the same irrigation level, the NUE gradually decreased with the increase of the N application rate, and the N_1_ treatment had the largest NUE, which was 11.22% - 35.02% (first cut) and 11.87% - 23.98% (second cut) higher than that in the N_4_ treatment. There were no differences between the three irrigation levels under the same N application rate. The W_1_N_1_ treatment had the maximum average NUE (2.57 kg/kg) of the two cuts, and the W_3_N_4_ treatment had the minimum value (1.77 kg/kg) (**Figures 4A, C**).

Under the same irrigation level, there were difference in the PFPN between the N treatments, and the PFPN decreased with the increase of N application rate. The PFPN in the N_2_, N_3_, and N_4_ treatment were 40.29 - 86.80% (first cut) and 38.06% - 73.85% (first cut) lower than that in the N_1_ treatment. Under the same nitrogen application rate, the PFPN first increased with the increase of irrigation volume and then stabilized. The W_1_N_1_ treatment had the maximum average PFPN (53.11 kg/kg) for the two cuts (**Figures 4B, D**).

### 3.4 The crude ash and crude protein content of alfalfa under different water and nitrogen supply conditions

**Figure 5:**
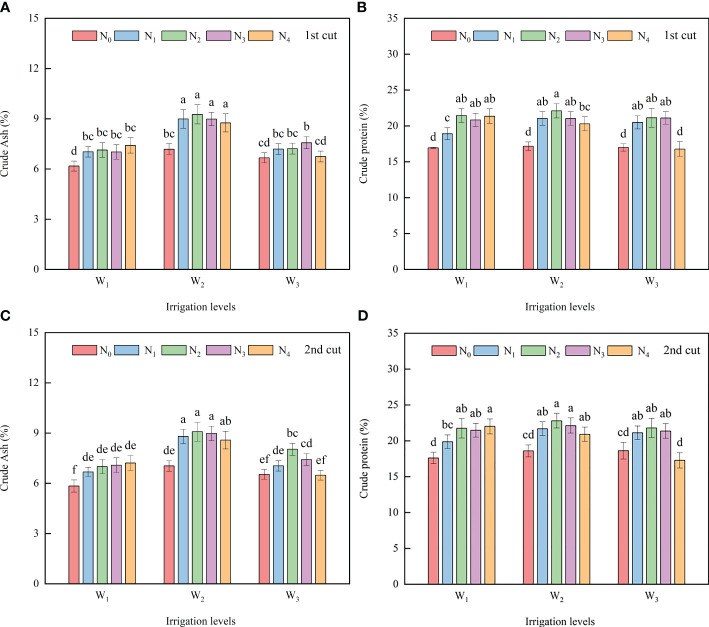
Effects of different irrigation volumes and nitrogen application rates on crude ash **(A, C)** and crude protein content **(B, D)** in alfalfa. Different letters on top of the bar denote significant difference at *p* < 0.05 (LSD test). Different lowercase letters indicate significant differences between treatments at *p* < 0.05 (Tukey test). The processing abbreviations are the same as those described in **Figure 3**.

At the W_1_ and W_2_ levels, the ASH in the N_1_, N_2_, N_3_, and N_4_ treatments had no difference, but were 11.96 - 22.50% (first cut) and 12.58% - 23.66% (second cut) higher than that in the N_0_ treatment. At the W_3_ level, the ASH first increased and then decreased with the increase of N application rate, and there was no difference between N_1_, N_2_, and N_3_ treatment. Under the same N application rate, the ASH at the W_2_ level was higher than that at the W_1_ and W_3_ levels. The W_2_N_1_, W_2_N_2_, and W_2_N_3_ treatments had a higher average ASH of the two cuts, and there was no difference in ASH between the three (**Figures 5A, C**).

At the same irrigation level, the CPC first increased and then decreased with the increase of N application rate. The CPC in the N_1_, N_2_, and N_3_ treatments had no difference, but were 10.31% - 22.31% (first cut) and 11.44% - 18.40% (second cut) higher than that in the N_0_ treatment. Under the same N application rate, except for the W_3_N_4_ treatment, the CPC in other N treatments was not affected by the irrigation volume. At each irrigation level, the N_1_, N_2_, and N_3_ treatments had a higher average CPC of the two cuts than other N treatments. Besides, there was no difference in the CPC between the three irrigation levels (**Figures 5B, D**).

### 3.5 Economic benefits under different water and nitrogen supply conditions

The annual gross profit ranged from 1450 to 3064 USD/ha, and the maximum value was 52.68% higher than the lowest value. At the same irrigation level, the net income first increased and then decreased with the increase of N application rate. The net income in the W_2_N_2_ treatment was the highest (1737 USD/ha), and that in the W1N0 treatment was the lowest (212 USD/ha) (**Table 2**).

**Table 2:** Economic benefits of alfalfa cultivation under different irrigation volumes and nitrogen application rates (USD/ha).

Treatment		Water cost	Fertilizer cost	Cost of cultivation	Gross profit	Economic benefit
Irrigation	Fertilization					
W_1_	N_0_	55	172	1011	1450	212
	N_1_	55	205	1011	1893	621
	N_2_	55	239	1011	2169	864
	N_3_	55	272	1011	2324	986
	N_4_	55	306	1011	1824	452
W_2_	N_0_	77	172	1011	1984	724
	N_1_	77	205	1011	2687	1393
	N_2_	77	239	1011	3064	1737
	N_3_	77	272	1011	2660	1299
	N_4_	77	306	1011	2425	1031
W_3_	N_0_	98	172	1011	2447	1165
	N_1_	98	205	1011	2711	1396
	N_2_	98	239	1011	2818	1469
	N_3_	98	272	1011	2851	1469
	N_4_	98	306	1011	1967	552

### 3.6 Coupling effects of water and N fertilizer on alfalfa yield, CWP, PFPN, quality, and economic benefits

The irrigation volumes had a significant impact on the yield, CWP, IWP, PFPN, ASH, NUE, and CPC of alfalfa. The N application rates had a significant impact on the yield, CWP, IWP, NUE, PFPN, and CPC, but had no impact on ASH. The water-N interaction had a significant impact on the IWP, PFPN, ASH, CPC, and CWP of alfalfa, but had no impact on NUE (**Tables 1**, **3**).

**Table 3:** Effects of different irrigation volumes and nitrogen application rates on the crop water productivity, irrigation water productivity, nitrogen use efficiency (NUE), partial factor productivity from applied N, crude ash content, crude protein content, and yield of alfalfa.

	Crop water productivity	Irrigation water productivity	Nitrogen use efficiency	Partial factor productivity from applied N	Crude ash content	Crude protein content
Irrigation	**	**	*	**	**	*
Nitrogen	**	**	**	**	ns	**
Irrigation × Nitrogen	*	**	ns	**	**	**

It was difficult to maximize yield, CWP, PFPN, CPC, and economic benefits simultaneously (**Table 4**). Therefore, it is necessary to further explore the optimal combination of water and nitrogen.

**Table 4:** The corresponding irrigation volumes and nitrogen application rates for maximum yield, crop water productivity, partial factor productivity from applied N, crude protein, and economic benefits.

Dependent variable Y	Regression equation	Y max	W/(mm)	N/(kg/ha)
Yield/Y_1_	Y_1_ = -10.1726 + 0.0524133W - 0.0000405778W^2 + ^0.0405016N - 0.0000254222WN - 0.0000853757N^2^	8.5	599.5	147.9
Crop water productivity/Y_2_	Y_2_ = -2.3501 + 0.0732867W - 0.0000752889W^2 + ^0.0823925N - 0.0000461778WN - 0.000184423N^2^	20.5	434.9	168.9
Partial factor productivity from applied N/Y_3_	Y_3_ = -18.3875 + 0.29835W - 0.000226W^2^ - 0.279407N - 0.000216267WN + 0.000627852N^2^	51.7	573.0	75.0
Crude protein/Y_4_	Y_4 = _4.948 + 0.0447W - 0.0000368889W^2 + ^0.0745967N - 0.0000519556WN - 0.000135407N^2^	22.5	476.2	184.1
Net return/Y_5_	Y_5_ = -33917.7 + 129.983W - 0.101436W^2 + ^98.0749N - 0.0636WN - 0.213381N^2^	11762.5	596.5	140.9

**Figure 6:**
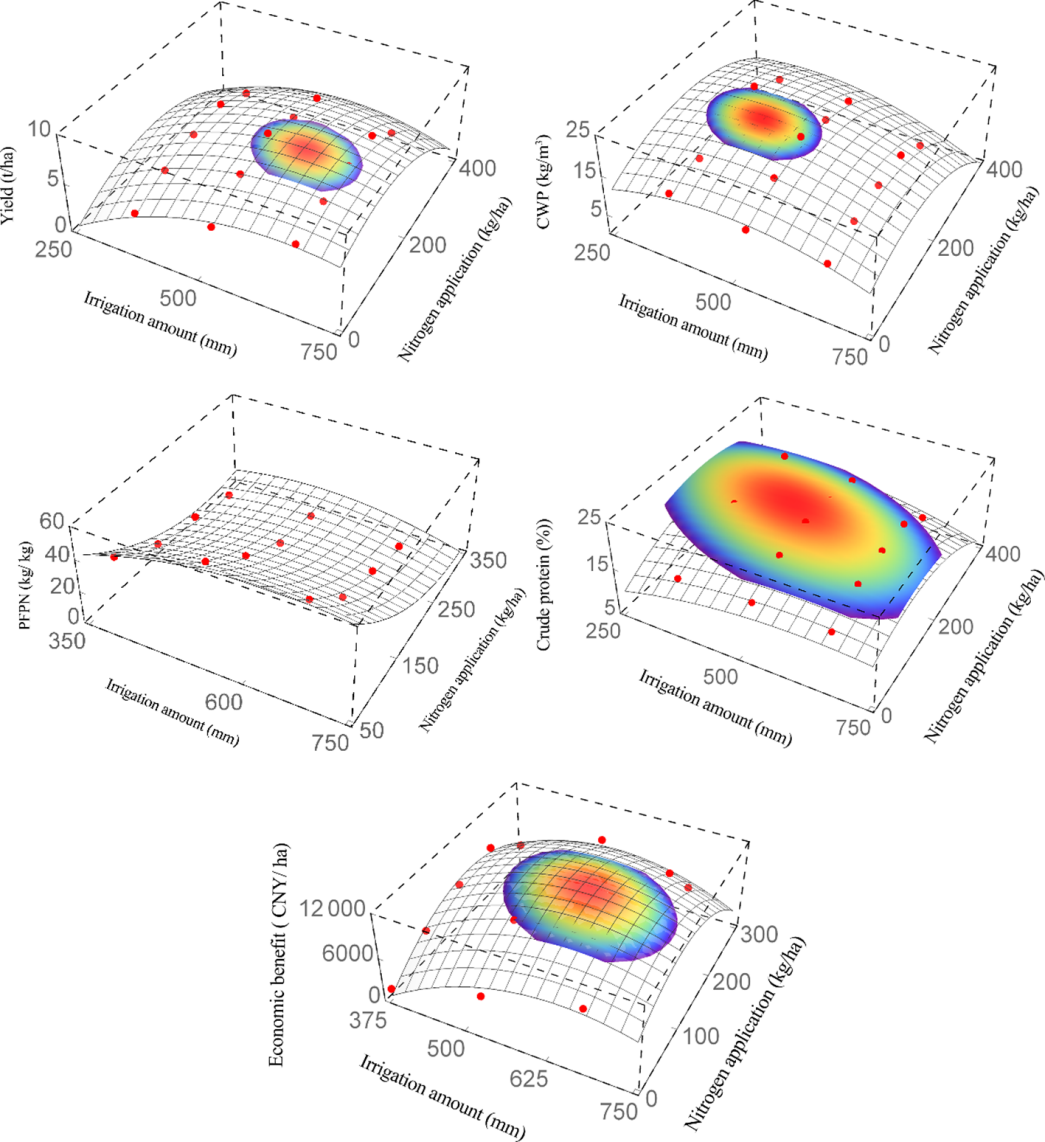
Relationship between alfalfa yield, crop water productivity (CWP), partial factor productivity from applied N (PFPN), crude protein content (CPC), economic benefits and water/nitrogen inputs.

The coupling effects of water and nitrogen on alfalfa yield, CWP, CPC, and economic benefits were convex, and the effect on PEPN was concave (**Figure 6**). Irrigation rates for maximum yield, CPC and economic benefits were similar, as were nitrogen application rates, but obviously different from those for CWP and PFPN. Further analysis at the 95%, 90%, 85%, 80%, and 75% confidence intervals showed that the overlap between maximum yield, CPC, and economic benefits was small at both 95% and 90% confidence intervals. There was a certain overlap between maximum yield, CPC, and economic benefits at both 80% and 75% confidence intervals, but the deviations of the indicators from the extreme value were large. Finally, it was found that it was acceptable to have a confidence interval greater than or equal to 85%, and the optimal solution was obtained. When the annual irrigation volume and N application rate were 473 ~ 601 mm (69.8 ~ 88.7% of ET_C_) and 145 ~ 190 kg/ha, respectively, the yield, CPC, and economic benefits could reach the optimal values (≥ 85%) (**Figure 6**).

## 4 Discussion

Plant height, branch number, and LAI play a crucial role in alfalfa yield formation, and these growth traits vary depending on cultivar, growth environment, and field management (Du et al., 2015; Ibrahim and Majid, 2018). In this study, alfalfa yield did not reach the maximum at the highest irrigation and N rates, and similar results were found in plant height, branch number, and LAI. Besides, the moderate irrigation and N application rates yielded the highest values (**Table 1**). This may be due to that too little or too much soil moisture can induce nutrient competition between vegetative and reproductive organs, affecting crop yields (Lodhi et al., 2014). Besides, improper N application may inhibit root growth and development, affect crop moisture and nutrient uptake, and therefore reduce yield (Liu et al., 2018). It was also found that alfalfa yield had a positive correlation with plant height, branch number, and especially LAI (**Figure 2**). This indicates that the increase in alfalfa yield is mainly due to the increase of LAI, and the increase in the number of leaves per unit area improves the efficiency of alfalfa photosynthesis, which in turn increases the yield of alfalfa.

Soil moisture and nutrient availability directly determines the nutrient absorption of plants, affects the nutrient content in different parts of plants, and ultimately affects crop yield and quality (Lv et al., 2023). In this study, when the irrigation volume was 75% of ET_C_ and the N application rate was 150 kg/ha, the CWP reached the maximum (21.35 kg/m^3^). This may be due to the fact that appropriate deficit irrigation and N supply is conducive to improving plant water and fertilizer use efficiency, promoting root development and canopy growth, and thus increasing alfalfa CWP and yield (Al-Gaadi et al., 2017; May et al., 2022). However, too low and too high N application rate affect the water uptake and utilization, and then reduce the water use efficiency (Xu et al., 2023). This study results showed that under the same irrigation level, there was a negative correlation between NUE, PEPN, and N rate, and the PEPN value under moderate irrigation level (W2) was significantly higher than that under low irrigation level (W1). This indicates that proper irrigation can not only increase yield, but also promote N absorption by alfalfa. It was also found that too little irrigation reduced the PEPN of alfalfa. This may be due to the weakening of nitrogen mineralization under insufficient water supply, as well as the detriment of N transport to the root system, thus affecting plant N uptake and utilization (Saini, 2017). In addition, it was found that PEPN was significantly affected by irrigation and N application, and fertilization had a greater effect on NUE than irrigation. This further suggests that optimizing water and N supply can improve alfalfa N use efficiency, thereby increasing productivity (Kang et al., 2023).

The quality of alfalfa is affected by both soil water and nutrients (Filho et al., 2018). Previous studies have shown that proper water and N supply can improve crop quality while maintaining yield (Kaplan et al., 2019; Kamran et al., 2023). This study found that at the same irrigation level, the ASH was not significantly affected by N application rate, but too low and too high N application rates reduced the CPC. The ASH was higher under moderate irrigation level (W2), and the CPC was not significantly affected by the irrigation volume. This indicates that N application rate has a greater effect on CPC than irrigation volume, and appropriate deficit irrigation is beneficial to increase the ASH of alfalfa by increasing the use of soil nutrients (Wu et al., 2010).

Appropriate amount of water and fertilizer input can improve crop water and fertilizer use efficiency to a certain extent, save costs, and obtain the optimal yield and economic benefits (Guo et al., 2022). In this study, the net income was the largest under the W_2_N_2_ treatment, increasing by 87.80% compared with the lowest value obtained under the W_1_N_0_ treatment. This increase in the net income in the W_2_N_2_ treatment is mainly due to that yield increase leads to lower average fixed cost. In summary, although the yield in the W_3_N_3_ treatment was highest, the plant height, branch number, LAI, CWP, NUE, CPC, and economic benefits were lower than those in the W_2_N_3_ treatment. Therefore, the W_2_N_2_ treatment (irrigation volume: 75% of ET_C_; N application rate: 150 kg/ha) is the optimal water and N fertilizer combination for alfalfa cultivation under subsurface drip irrigation in northwest China. Compared with the commonly adopted irrigation and N application rate (W_3_N_3_), the W_2_N_2_ treatment can save 25% water and reduce N input by 33%.

Yan et al. (2021) used multiple regression analysis to comprehensively evaluate the yield, N accumulation, and economic benefits of drip-irrigated spring maize. Wang et al. (2018) used multiple regression analysis and spatial analysis methods to analyze the effects of water and N input on cucumber yield, CWP, vitamin C content, soluble sugar content, NUE, and PFPN, and found that the maximum value of each index at the 90% confidence interval was acceptable, corresponding to an irrigation amount range of 124 ~ 151 mm and a nitrogen application rate range of 318 ~ 504 kg/ha. In this study, a multi-objective optimization model was established to analyze the relationship between alfalfa growth, yield, quality, economic benefits, and water/N fertilizer input. It was found that the yield, CPC, and economic benefits reached more than 85% of the maximum values when the irrigation volume was in the range of 473 ~ 601 mm and the N application rate was in the range of 145 ~ 190 kg/ha. Therefore, this can be used as the optimal water and fertilizer management strategy for the sustainable production of alfalfa under subsurface drip irrigation in the study area.

## 5 Conclusion

Alfalfa yield, crude ash content, and partial factor productivity from applied N increased significantly at the irrigation level 75% of ET_C_ compared with those at other irrigation levels. Alfalfa plant height, branch number, leaf area index, yield, crop water productivity, irrigation water productivity, crude protein content, and economic benefits reached a maximum when the N application rate was 150 kg/ha or 225 kg/ha. Therefore, the appropriate amount of water and fertilizer input can increase alfalfa resource use efficiency, yield, quality, and economic benefits, and the W_2_N_2_ treatment is the best water and N fertilizer combination for alfalfa cultivation under subsurface drip irrigation, which can save 25% water and reduce N input by 33% compared with the commonly adopted irrigation and N application rate (W_3_N_3_). Besides, this study determined that when the irrigation volume was 69.8 ~ 88.7% of ET_C_ and the N application rate was 145 ~ 190 kg/ha, the yield, crude protein content, and economic benefits of alfalfa reached the highest values at the confidence interval ≥ 85%. This study is of great significance for the water and nitrogen management in alfalfa cultivation under subsurface drip irrigation in northwest China and other arid and semi-arid areas.

## Data availability statement

The original contributions presented in the study are included in the article/**Supplementary Files**, further inquiries can be directed to the corresponding author/s.

## Author contributions

HM: Writing – original draft. PJ: Writing – review & editing. XZ: Writing – review & editing. WM: Writing – review & editing. ZC: Writing – review & editing. QS: Writing – review & editing.
