# Supplementary material for: Effects of nitrogen fertilization combined with subsurface irrigation on alfalfa yield, water and nitrogen use efficiency, quality, and economic benefits
Source: Front Plant Sci. 2024 Jan 29;15:1339417. doi: 10.3389/fpls.2024.1339417 (PMC10859442; doi:10.3389/fpls.2024.1339417)

**Figure S1.** Average daily temperature and precipitation during alfalfa growing season and evapotranspiration of reference crops (ET_0_).


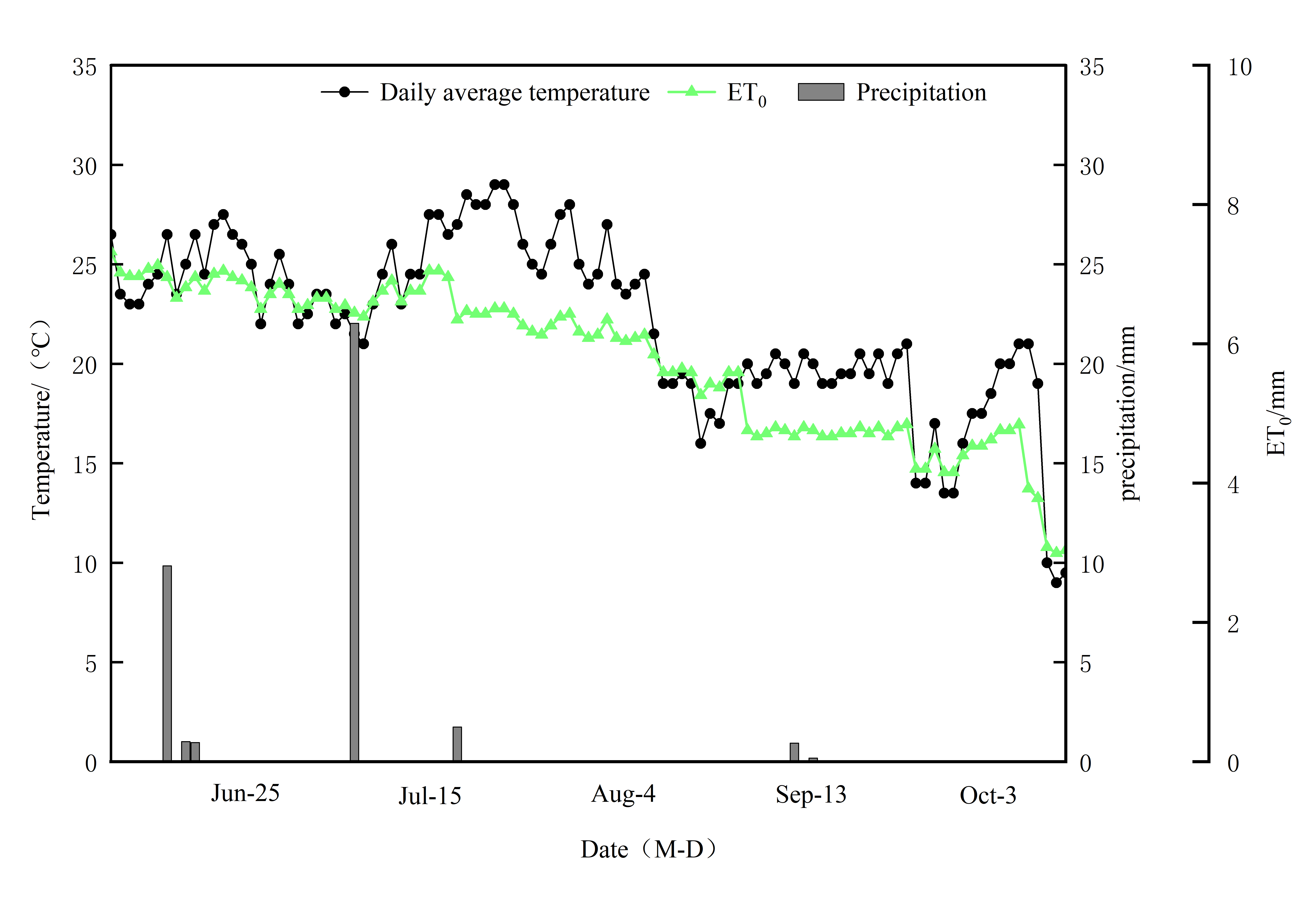

Supplement: Supplementary file 1 [file DataSheet_1.docx]
